# Supplementary material for: Rapid turnover of life-cycle-related genes in the brown algae
Source: Genome Biol. 2019 Feb 14;20:35. doi: 10.1186/s13059-019-1630-6 (PMC6374913; doi:10.1186/s13059-019-1630-6)
Supplement: Supplementary file 3 — Protein alignments, corresponding Gblocks html file and CDS sequences for the lineage conserved gametophyte-biased genes. (ZIP 2109 kb) [file 13059_2019_1630_MOESM3_ESM.zip › Con_GA_alignments/Ec-12_001260.aa_ali.fasta-gb.htm]

Ec-12\_001260.aa.fasta.trans.aa\_ali.fasta


## Gblocks 0.91b Results

Processed file: **Ec-12\_001260.aa.fasta.trans.aa\_ali.fasta**  
Number of sequences: **2**  
Alignment assumed to be: **Protein**  
New number of positions: **153** (selected positions are underlined in blue)

```
                         10        20        30        40        50        60
                 =========+=========+=========+=========+=========+=========+
Ec-12_001260     MGEPRISLFKCRDPEDVMSLARCLRRCKSTPALSYTAASLLSKTLTARATRLESTASKAR
MSTRG.33052.1    ------------------------------------------------------------
                                                                             


                         70        80        90       100       110       120
                 =========+=========+=========+=========+=========+=========+
Ec-12_001260     LTAEKEAYRRATVEKRRRALGPKNPSDFMSQTLRDREKARENNRLRAERRRPGTPVLAES
MSTRG.33052.1    ------------------------------MIMRTKCTTRSNENVE---K------VVGT
                                                                             


                        130       140       150       160       170       180
                 =========+=========+=========+=========+=========+=========+
Ec-12_001260     AAVCRRGTRRAKQGPLSEISLQFRFGESAHDNRSTHSSLGGSLAHGSGAGPPQRTEQTST
MSTRG.33052.1    QALCVP---------------QCRF----HGERSAR--LFC----------TCLQERSST
                                                                        #####


                        190       200       210       220       230       240
                 =========+=========+=========+=========+=========+=========+
Ec-12_001260     PHPRSTPMPDCVGLEDDDDIHVLLQRAYSSSSSIRSE----SSGFSGKPK----PTSGQS
MSTRG.33052.1    HRPDVIPTSDRVTLEDDADVRVLLDRTQSSRSCTPSEKVPRPATSCGRRRESALSASSGP
                 #####################################                   ####


                        250       260       270       280       290       300
                 =========+=========+=========+=========+=========+=========+
Ec-12_001260     YGRSHCTAESPLLQGKPSAAACKGSVRRPHTAGATATARSFDGRKFSPADVAQMRREILI
MSTRG.33052.1    FARPRARAEPSPVPGSSSAQAHEAFSRRPHTAGAYRREIS---SEIDTSDMFRLRREMLI
                 ########################################         ###########


                        310       320       330       340       350       360
                 =========+=========+=========+=========+=========+=========+
Ec-12_001260     RSHGEKRHLHLYPAEPFETTAMGRKARCRTPGRKAELMPWMPAKTTDSLDRLQRSRMLMN
MSTRG.33052.1    RSHGEGRNANLFAAKQFETTAMGRKK-CDH---QPRLTPWIPASTLAGLDRVKRSRMLMN
                 #########################           ########################


                        370
                 =========+===
Ec-12_001260     QRPVVAKLH-AWT
MSTRG.33052.1    QRPVVSKIPSSWA
                 #######
```

```
Parameters used
Minimum Number Of Sequences For A Conserved Position: 2
Minimum Number Of Sequences For A Flanking Position: 2
Maximum Number Of Contiguous Nonconserved Positions: 8
Minimum Length Of A Block: 10
Allowed Gap Positions: None
Use Similarity Matrices: Yes
```

```
Flank positions of the 4 selected block(s)
Flanks: [176  217]  [237  280]  [290  325]  [337  367]  

New number of positions in Ec-12_001260.aa.fasta.trans.aa_ali.fasta-gb:  153  (41% of the original 373 positions)
```
